# Simplified Models of Vector Control Impact upon Malaria Transmission by Zoophagic Mosquitoes

**DOI:** 10.1371/journal.pone.0037661

**Published:** 2012-05-31

**Authors:** Samson S. Kiware, Nakul Chitnis, Sarah J. Moore, Gregor J. Devine, Silas Majambere, Stephen Merrill, Gerry F. Killeen

**Affiliations:** 1 Biomedical and Environmental Thematic Group, Ifakara Health Institute, Ifakara, Tanzania; 2 Department of Mathematics, Statistics, and Computer Science, Marquette University, Milwaukee, Wisconsin, United States of America; 3 Department of Epidemiology and Public Health, Swiss Tropical and Public Health Institute, Basel, Switzerland; 4 University of Basel, Basel, Switzerland; 5 London School of Hygiene and Tropical Medicine, London, United Kingdom; 6 Vector Group, Liverpool School of Tropical Medicine, Liverpool, United Kingdom; University of Hong Kong, Hong Kong

## Abstract

**Background:**

High coverage of personal protection measures that kill mosquitoes dramatically reduce malaria transmission where vector populations depend upon human blood. However, most primary malaria vectors outside of sub-Saharan Africa can be classified as “very zoophagic,” meaning they feed occasionally (<10% of blood meals) upon humans, so personal protection interventions have negligible impact upon their survival.

**Methods and Findings:**

We extended a published malaria transmission model to examine the relationship between transmission, control, and the baseline proportion of bloodmeals obtained from humans (human blood index). The lower limit of the human blood index enables derivation of simplified models for zoophagic vectors that (1) Rely on only three field-measurable parameters. (2) Predict immediate and delayed (with and without assuming reduced human infectivity, respectively) impacts of personal protection measures upon transmission. (3) Illustrate how appreciable indirect communal-level protection for non-users can be accrued through direct personal protection of users. (4) Suggest the coverage and efficacy thresholds required to attain epidemiological impact. The findings suggest that immediate, indirect, community-wide protection of users and non-users alike may linearly relate to the efficacy of a user’s direct personal protection, regardless of whether that is achieved by killing or repelling mosquitoes. High protective coverage and efficacy (≥80%) are important to achieve epidemiologically meaningful impact. Non-users are indirectly protected because the two most common species of human malaria are strict anthroponoses. Therefore, the small proportion of mosquitoes that are killed or diverted while attacking humans can represent a large proportion of those actually transmitting malaria.

**Conclusions:**

Simplified models of malaria transmission by very zoophagic vectors may be used by control practitioners to predict intervention impact interventions using three field-measurable parameters; the proportion of human exposure to mosquitoes occurring when an intervention can be practically used, its protective efficacy when used, and the proportion of people using it.

## Introduction

Indoor residual spraying (IRS) and long-lasting insecticidal nets (LLIN) dramatically reduce malaria transmission [1]. Both approaches exceed the benefits of personal protection and provide even greater levels of community-wide protection for users and non-users alike once reasonably high coverage is achieved (30%–60%) [2]–[3]. High demographic coverage of humans 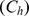 can dramatically reduce the density, longevity and infection prevalence of mosquito species that primarily feed indoors (endophagic) upon humans (anthropophagic) such as *Anopheles gambiae* and *An. funestus* from sub-Saharan Africa [4]–[6] or *An. punctulatus* and *An. koliensis* from the Pacific [7]. The massive importance of community-level transmission suppression for realizing the full potential of both IRS [8] and LLINs [2] using contact insecticides is well established and reflected in global universal coverage targets for these interventions [9]. Also, vector population modification by LLINs and/or indoor residual spraying (IRS) [4]–[5], [10]–[12], has been observed since the Global Malaria Eradication Programme (GMEP) was initiated in the 1950s. For example, *An. funestus* was replaced by *An. rivulorum* and/or *An. parensis* following the introduction of IRS on at least three distinct occasions in South Africa, Kenya and Tanzania [13]–[16].

However, mosquitoes which feed upon animals (zoophagic) are primary malaria vectors in many tropical countries [17]–[18] and can dominate residual transmission in settings where high demographic coverage of LLIN or IRS has successfully suppressed previously predominant, anthropophagic species [4]–[5], [10], [12]–[13].

While LLINs confer personal protection against any mosquitoes attempting to bite while they are in use, it remains unclear whether they confer community-level protection against zoophagic vectors that feed only occasionally upon humans. We therefore extended a previously published static malaria transmission model [6] and applied it to explain how immediate and delayed impacts of personal protection measures can be predicted using three potentially field measurable parameters. In addition, we simplified this model formulation by expressing malaria transmission and control in terms of a baseline human blood index [19]. Also, the model was used to assess the likely extent and mechanism of the community-level impact of such personal protection measures upon human malaria exposure for the zoophagic vectors that are primary vectors in many parts of the world [4], [10], [18] and will increasingly dominate transmission in the future [12], [20]. We also contrast these impacts and underlying mode of action with those of the anthropophagic species that have been the overwhelming focus of malaria research and control to date.

## Methods

### Model Description

We extended a static malaria transmission model [6] to explore the dependence of malaria transmission and control upon baseline human blood index before any intervention is introduced. Specifically, the impact of personal protection measures such as LLINs, IRS, insecticide-treated clothing or repellents upon the baseline malaria transmission intensity was compared in a range of vector behaviour scenarios.

### Simulating Malaria Transmission and Control as a Function of Mosquito Host Preference

Before describing how the model simulations were performed, we first present the basic input parameters and their definitions, equations and derived parameters, output from the model, description of simplified models for very zoophagic vectors, and the expression of malaria transmission and control as a function of baseline human blood index.

### Model Basic Input Parameters and Definitions

Several subscripts are used in this model; 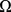 denotes an intervention package scenario consisting of a specific coverage, 0 for a baseline condition with no intervention,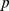 for protected or 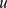 for unprotected humans 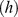, and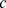 for cattle or other animals. Demographic or crude coverage is defined as a proportion of people using a personal protection measure as estimated in a standardized malaria indicator surveys 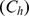 [6]. Another important input is the proportion of daily exposure that a non-user would typically experience at times when a user would normally use such a personal protection measure 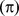. In other words, this is the maximum proportion of human exposure to mosquitoes that can be directly prevented through a personal protection by using a given measure. This is a broader definition than used previously when the term was described as the proportion of human exposure that occurs indoors while asleep at times when LLINs can be used 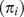 [21]. This more generalized definition allows the incorporation of other personal protection interventions such as insecticide-treated clothing and repellents which can also be used outdoors. Recently, several authors [21]–[23] have described and discussed the importance and measurement of 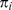, but the concept was also discussed during the GMEP era [24]–[25] when the difficulty of controlling exophagic or exophilic vectors was described in Africa [21], [26], Asia [27], and the Americas [25]. We also introduce host-encounter rate 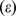 which is the rate at which a single host-seeking mosquito encounters a given single host. The notations,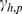,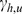, and 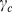 represent probability of attacking encountered protected humans, unprotected humans and cattle, respectively. Whereas,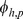,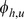, and 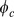 represent mosquito feeding probability upon protected humans, unprotected and cattle respectively. The mean attack availability of individual cattle 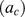 is the rate at which a single mosquito encounters and then attacks a single cow whereas the mean attack availability of an individual unprotected 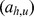 human, is the rate at which a single mosquito encounters and then attacks a such single person of either protection status [6]. Mortality probability upon attacking a protected or an unprotected human or cow are denoted by 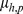, 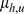, and 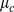, respectively. 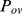 denotes the survival probabilities during host-seeking and ovipisition site-seeking, which are assumed to be equal. 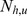 and 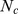 are the population sizes of unprotected humans and cattle, respectively. The subscripts and the basic parameters presented here are also defined in table 1 with their dimensions listed for a quick reference.

**Table 1:** Definition of basic parameters.

Symbol	Definition and explanation	Dimension
ε	Host-encounter rate: rate at which a single host-seeking mosquito encounters agiven single hosts.	One
ε_h_, ε_c_	Human and cattle encounter rate respectively.	Per Time
ϕ_h,u_	Probability that a mosquito which attacks an unprotected human will successfullyfeed upon that host.	One
ϕ_h,p_	Probability that a mosquito which attacks protected human will successfully feed upon that host.	One
_γh,p, γh,u, γc_	represent probability of encountering protected, unprotected human and cattle respectively.	
_Nh, Nh,p, Nh,u_	Number of people, protected and unprotected	Human
_Nc_	Number of cattle	Animal
C_h_	Demographic or crude coverage: Proportion of people using a personal protectionmeasure as estimated in a standardized malaria indicator surveys.	One
µ_h,u_	Mortality probability upon attacking an unprotected human.	One
µ_h,p_	Mortality probability upon attacking an protected human	One
µ_c_	Mortality probability upon attacking a cattle	One
π_i_	The proportion of normal exposure to mosquito bites upon humans lacking LLINs,which occurs indoors at times when nets would normally be in use.	One
π	The maximum proportion of human exposure to mosquitoes that can bedirectly prevented through personal protection by using a given intervention	One
P_ov_	The survival probabilities during host seeking and ovipisition site-seekingassumed to be equal	1/exp(Time)

The subscripts used are given in bracket; human (h), protected (p), unprotected (u), cattle (c), a baseline condition with no personal protection coverage (0), intervention package scenarios consisting of a specific coverage (Ω).

### Model Equations for Derived Parameters

We present equations from previous model [6] that are of important to this paper relating all derived parameters in terms of the basic parameters or other already derived parameters. Though these derived parameters are defined here, their definitions and dimensions are also presented in table 2.

**Table 2:** Definitions of the derived parameters.

Symbol	Definition and explanation	Units
C_h,p_	Protective coverage	One
a_c_	Mean availability of individual cow for attack: rate at which a single mosquitoencounters and then attacks a cow or pseudo-host.	Per time per animal
a_h_	Mean availability of individual human for attack: rate at which a single mosquitoencounters and then attacks a human or pseudo-host.	Per time per human
a_h,p_	Availability of individual protected human	Per time per protected human
a_h,u_	Availability of individual unprotected human	Per time per unprotected human
A, A_h_, A_c_	Total availability of all hosts, all humans and all cattle, respectively: rate at which asingle mosquito encounters, attacks upon these host sets	Per time
z, z_h_, z_c_	Mean availability of blood from all hosts, all humans and all cattle, respectively: rate at whicha single mosquito encounters, attacks and successfully feeds upon these host sets.	Per time
Z, Z_h_, Z_c_	Total availability of blood from all hosts, all humans and all cattle, respectively: rate at whicha single mosquito encounters, attacks and successfully feeds upon these host sets.	Per time
Q_h_	Human blood index: the proportion of all blood meals from all hosts which are obtained from humans.	One
Q_h,0_	The baseline human blood index in the absence of any protection measure	One
P_γ_	Probability of surviving host attack per feeding cycle	One
η_0_	Oviposition site-seeking interval; number of days a mosquito takes to findan oviposition site once it starts searching for it	Time
η_v_	Host seeking interval: number of days a mosquito takes to find and attack a vertebrate host	Time
P_f_	The survival rate per feeding cycle	Per time
f	Feeding cycle length: measured as the number of days it takes a singlemosquito to get from one blood feed to the next.	Time
E	Emergence rate of mosquito vector	Per time
β_h_	The total number of infectious bites on all humans	One
β	The total number of sporozoite infected bites in all hosts per mosquito lifetime	One
EIR	Entomological inoculation rate (mean number of infectious bites thatan average individual human receives per year).	Per time
EIR_h,Ω_	absolute EIR for an average community member in a given intervention scenario	Per time
EIR_h,u_	EIR for non-users	Per time
ψ_h,u_	The immediately relative exposure of non-users benefiting only from communal protection	One
g	Gestation interval: number of days a mosquito takes to digest a blood mealand return to searching for oviposition site.	Time
P^g^	Combined probability that a vector survives gestation	One
x	Mosquito age	Time
S_x_	The sporozoite infection prevalence of mosquitoes at each age	One
χ	Human infectiousness to mosquitoes: probability of a vector becoming infected per human bite.	One
ρ	Overall proportion of personal protection against mosquito bites provide by using a givenprotective measure.	One
ψ↑_h,u,Ω_	The immediate impact on vector population assuming a reduction of human infectivity.	One
P_f_^x/f^	Estimation of daily cycle and cumulative survival of mosquitoes up to each age (x).	One

The subscripts used are given in bracket; human (h), protected (p), unprotected (u), animals (c), a baseline condition with no personal protection coverage (0), intevention package scenarios consisting of a specific coverage (Ω).

### Protective Coverage and Baseline Human Blood Index

As previously [6], we define *de facto* protective coverage of humans 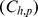 as being the product of crude coverage 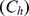, and the maximum proportion of human exposure to mosquitoes that can be directly prevented through personal protection by using a given intervention 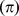;
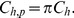
(1)


The mean availability 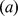 of any host of any species 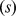 for mosquitoes to attack is the product of the rate at which individual vectors encounter that host 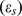 and the probability that, after this encounter, they will attack the host 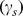;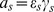 [28]. Thus, 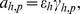 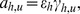 and 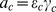 are mean attack availability of protected, unprotected human and cattle respectively. The mean availability of host blood 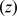 from a host of any species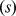 is the product of the rate at which individual vectors encounter this host 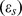
and the feeding probability upon that particular host 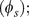 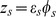 [28]. Thus, 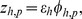 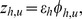 and 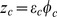 represent mean availability of blood from individual protected, unprotected human and cattle respectively.

The total availability of all hosts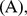 protected humans 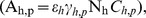 unprotected humans 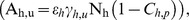 and all cattle 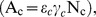 respectively, are the rates at which a single mosquito encounters, attacks upon these host sets [6]. These total availability parameters are related to each other and calculated in terms of basic individual availability and host population size parameters as follows [6];
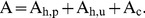
(2)


Similarly, the total availability of blood from all hosts, 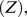 protected 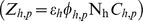 or unprotected 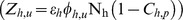 humans and all cattle 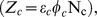 respectively is the rate at which a single mosquito encounters, attacks and successfully feeds upon these host sets [6] given by;
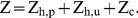
(3)


The human blood index is the proportion of all blood meals obtained from both protected and unprotect humans [19], and is calculated as a function of the total availability of blood from both categories of humans and the availability of alternative blood sources such as cattle and other animals [6]:
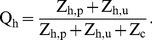
(4)


Changing the mean availabilities of protected humans 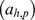 or unprotected humans 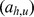 and cattle 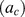 correspondingly change,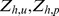 and 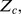 and therefore the the human blood index 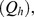
because 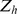 is directly related to 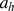 whereas 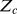 is directly related to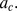 The baseline human blood index in the absence of any protection measure 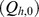
can be used to identify vector populations which are zoophagic in terms of both their innate host preferences and their ability to exploit locally common animal hosts. This is because low values represent mosquitoes that primarily feed on animals (zoophagic) while high values represent those that primarily feed on humans (anthropophagic). So, when 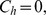 the baseline human blood index 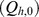 can be derived in terms of basic parameters as;
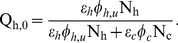
(5)


For predominantly animal-feeding mosquito [29], we assume that the mean encounter rate for humans 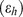 approaches zero, so that the same is correspondingly true of the mean attack availability of humans 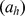 and the mean availability of human blood *per se* 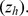 Therefore, the total attack availability of all humans 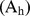 and the total availability of all human blood *per se* 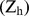 also approaches zero.

In equation 5, baseline human blood index goes to zero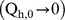 when either the denominator goes to infinity or the numerator goes to zero. The numerator can go to zero in three different ways; either when 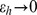 or 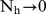 or 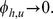 It is unrealistic that the denominator will go to infinity, or that 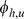
will go to 0, and it is of no interest to model malaria transmission in the situation where 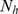 goes to zero. So, in the situations that are realistic and interesting, 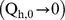
if and only if 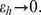 Hence, when we are interested in the situation 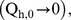 we can take the limit as 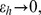
which biologically means a situation where mosquitoes are not attracted to human blood so the attractiveness or availability of human blood is close to zero. Therefore, the mean availability of individual humans 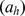 and the mean availability of blood from individual humans 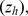 the total availability of all humans
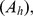
and the total availability of all humans blood 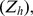 including both the protected and unprotected, all approach zero as well.

### Model Outputs

Malaria transmission intensity is often expressed in terms of the entomologic inoculation rate (EIR) which is a direct, field-measurable indicator of human exposure to bites of mosquitoes infected with transmissible sporozoite stage malaria parasites [30]–[31]. Thus, the primary outputs from the model were the absolute EIR for an average community member 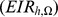 and the relative exposure for non-users to the baseline condition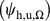 both in a given intervention scenario. To help understand how the impact of a personal protection measure mediated in a given scenario 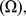 the impact upon vector population parameters, the survival rate per feeding cycle 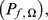 human blood index 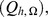 feeding cycle length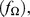 and emergence rate of adult mosquitoes 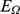 are plotted against 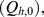 as intermediate secondary outputs that underlie EIR and changes in this primary outcomes.

We present equations from *Killeen et al* [6] necessary to define primary and secondary outputs in terms of basic or already derived parameters. The probability of surviving host attack per feeding cycle 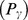 is a function of the probability of surviving one complete feeding cycle 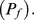 The oviposition site-seeking interval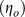 and the vertebrate host-seeking interval 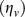 are both a function of feeding cycle length 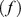 and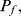 where both 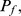 and 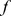
are functions of emergence rate of adult mosquitoes 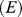 [6]. So, we first present equations of 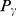
and the combined 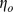 and 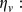


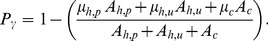
(6)

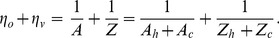
(7)


Hence, 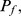 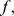
and 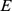 [6] are given as follows:
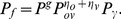
(8)

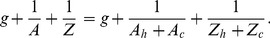
(9)

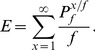
(10)Where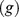 is gestation period and 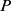 is the mean daily survival, 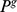
is the probability that a vector survives a single gestation, and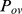 is the survival probability for the combined host seeking and ovipisition site-seeking intervals. Whereas, 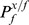 is the cumulative survival of mosquitoes up to a given age 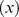, as previously described [6]. In all cases, impact is assessed in terms of changes in the parameters under a given scenario 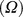 relative to a baseline with no protection measure (0): 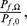 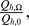 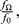 and 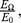 respectively.

The number of infectious bites on humans 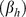 per mosquito life time is given by the product of human blood index and the sum of the products of the probabilities of surving and being infectious at each age [6];
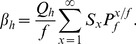
(11)Whereas, 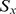 is the sporozoite infection prevalence of mosquitoes at each age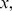 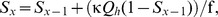 for 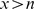 otherwise 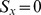 where, 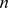 is the extrinsic incubation period, and 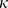 is population mean human infectiousness to mosquitoes; defined as the mean probability of a vector becoming infected per human bite.

Thus, absolute EIR for an average community member in a given intervention scenario 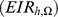 is given by [6];
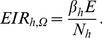
(12)


The relative exposure for non-users 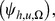
humans who are unprotected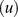 by the physical and chemical barrier of personal protection measure but may benefit from communal protection, in a given intervention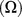 scenario is calculated as their predicted exposure 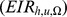 divided by their baseline exposure with no protection (0) measure 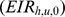
as;
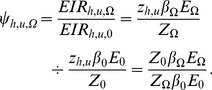
(13)Whereas, 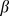 is the number of sporozoite infected bites in all hosts per mosquito lifetime 
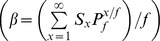
 calculated as equation 11 but ignoring 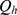 term [6].

### Simplified Models for Very Zoophagic Vectors

Initial simulations suggested closer examination of the underlying mechanisms through which personal protection mediates community-level protection against malaria transmission by very zoophagic mosquitoes. We specifically define very zoophagic vectors as those which are not merely zoophagic, such as *An. arabiensis* which readily feeds on both humans and cattle [32], but rather those which have a strong preference for animals and normally obtain 90% or more of their blood meals from animals 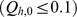. A useful example of such a vector species that can be considered very zoophagic is *Anopheles epiroticus* in the Mekong delta of Vietnam. This mosquito population has a >11-fold preference for cattle over humans [27], which allows us to simulate transmission by this species by adjusting the mean encounter rate for humans 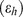 in proportion to this relative attack rate of cattle compared with humans [6], [28], [33], but which are otherwise equivalent to those described above for An. arabiensis [6]. It illustrates how mosquitoes exhibiting very high levels of zoophagy at population level 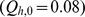 can mediate transmission intensities 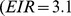 infectious bites per person per year) that are compatible with this mosquito’s status as a primary malaria vector in the region [34].

### Expressing Malaria Transmission and Control as a Simplified Function of Baseline Human Blood Index

We express the primary and secondary outputs in terms of human blood index 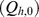, because it is one of the most important determinants of overall malaria transmission locally and globally [17], [19], [35]–[37]. For very zoophagic mosquito populations with low human blood indices 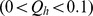 that are nevertheless sufficient to stably transmit malaria 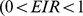 infectious bite per year per person); we are interested in a situation where 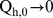 to illustrate the impact of a personal protection measure on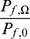,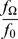, 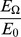, and 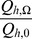.

Since 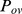 is constant, using equation 6 and 8 we can compute 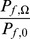 as 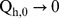 by taking the limit as 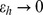, (so 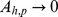,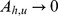, 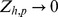, 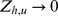) terms only with subscript 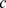 (for cattle) remain cancelling to 1;
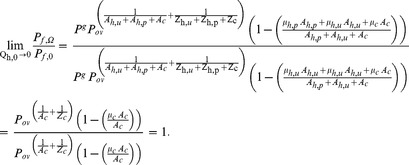
(14)


Using equation 9 the same approach can be applied for 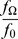 to get;
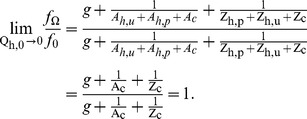
(15)We use equation 10 to drive 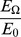 in the limit 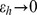
by rearranging equation 10 and then substituting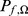,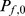,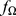, and 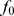 from equations 14 and 15 as follows;



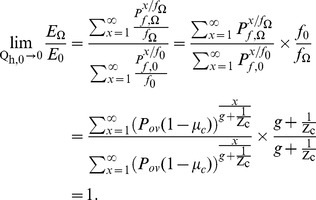
(16)The interpretation of equation 14, 15 and 16 is given in the result section. However, the limit for the other vector population parameter does not go to 1, indicating that human blood index is affected by personal protection measures against very zoophagic vectors that are nevertheless fractionally but sufficiently anthropophagic to put a lot of people at risk of malaria transmission. This allows much simpler models for both immediate impacts upon malaria transmission, with and without an assumed reduction of human infectivity in the longer term, to be derived that rationalize the reduced, but nevertheless useful, impacts of insecticidal personal protective measures upon zoophagic vectors. The explanation and interpretation of what happens to the overall impact on 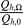 as 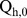 approaches zero for very zoophagic 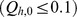 vectors, is provided in the results section.

### Simulated Scenarios

The full possible range of host preference for mosquitoes was simulated by modifying field estimates for cattle and human encounter rate, 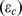 and 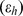 respectively, by beginning with values typical of a mosquito such as *An. Arabiensis,* which is both anthropophagic and zoophagic [33], [35], [38]–[39]. The value for 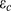 was tuned down to zero to mimic highly anthropophagic African vectors like *An.gambiae* [33], while 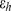 was tuned down towards zero to mimic zoophagic mosquitoes like *An.quadriannulatus* [38], [40] and other Anophelines that only occasionally feed on humans [38], [41]–[42]. While *An.gambiae, An.arabiensis and An.quadriannulatus* come from a single African species complex (*An.gambiae sensu lato*); they span the full range of host choice preferences exhibited by anophelines world-wide. Although *An.gambiae* typically feeds almost exclusively upon humans, and has historically been the most important vector of malaria in the world [43], *An.arabiensis* is as likely to attack cattle as humans and is a correspondingly less potent but nevertheless significant primary vector [43]–[45]. By comparison, *An.quadriannulatus* is thought to rarely feed upon humans and transmit little, if any malaria, despite being readily infected by *Plasmodium falciparum* [46]. *An.arabiensis* is a useful intermediate example because this species has been well studied, feeds readily upon both humans and animals [32], [47], and has proven relatively resilient to control with IRS and LLINs [40].

The first scenario was simulated with no intervention by setting 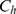 to 0, whereas, the intervention scenarios 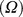 were simulated by setting 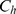 for an unspecified personal protection measure to the assumed high coverage levels of 0.8, equivalent to the Roll Back Malaria targets for LLIN coverage of all age groups, with a very high proportion of human exposure to mosquitoes occurring when that protection measure can practically be used 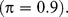


The model was implemented with a range of values of 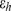 ranging from a maximum of 1.7×10^−3^ and then decreasing to 1.1×10^−4^ encounters per day per host-seeking vector per unprotected human, with 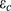 increasing from 0 up to 1.7×10^−3^ encounters per day per host-seeking vector per cow. The default value of 1.7×10^−3^ encounters per day per host-seeking vector per unprotected human, at which these two ranges coincide, is used because it is an intermediate value between field measures for 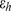 of 1.3×10^−3^ and for 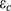 of 2.1×10^−3^ encounters per day per host-seeking for *An. arabiensis* [2]. 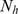 and 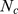 were assumed equal (1000 for each) in all simulations, leading to 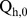 values ranging from 0.03 to 1.00.

## Results

**Figure 1:**
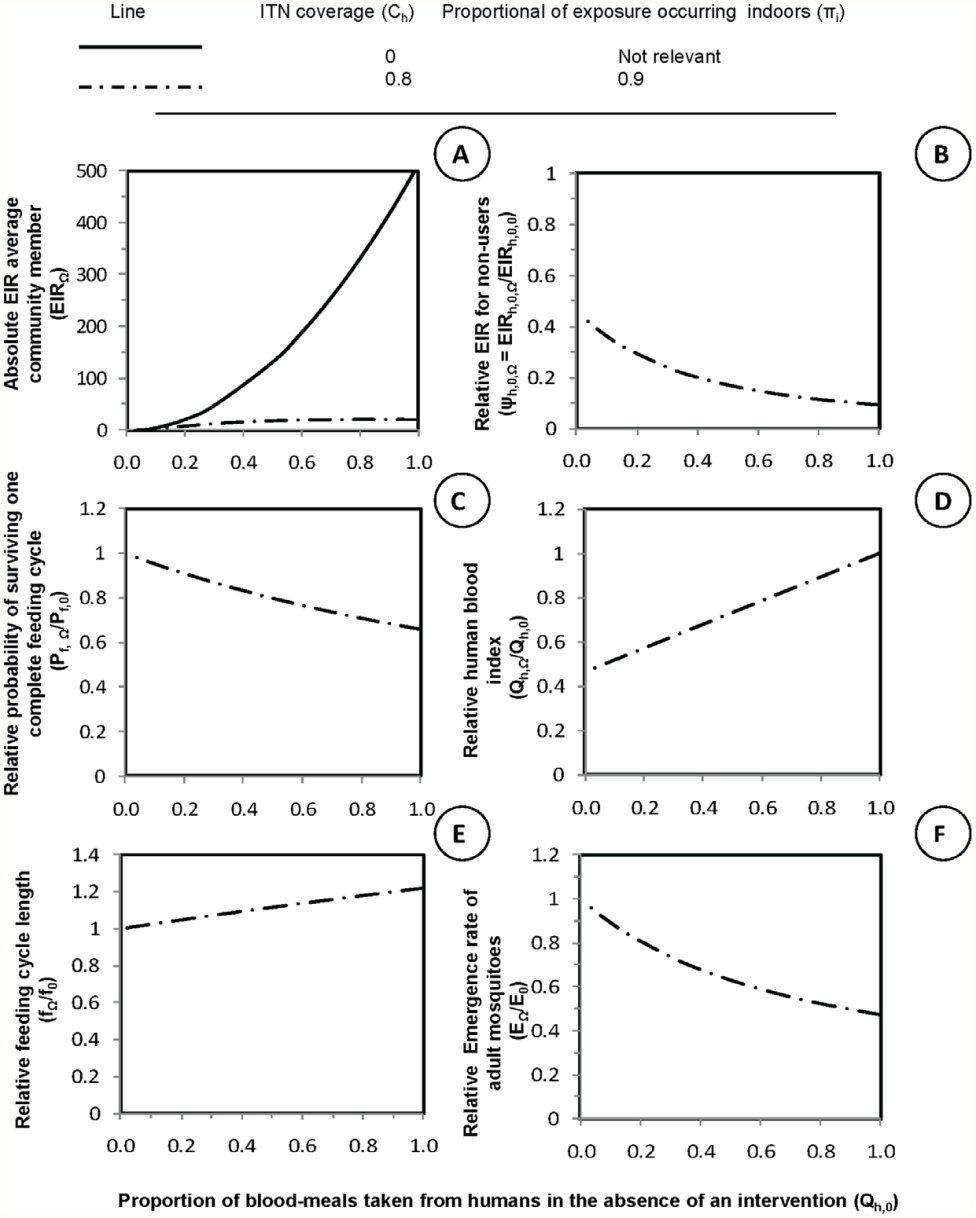
The impact of long lasting insecticide treated nets (LLINs) upon malaria vector population parameters. Malaria vector population parameters, transmission intensity, and the impact of personal protection interventions upon them under a range of values for the proportion of blood meals obtained from humans 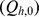. In all panels, the x-axis is the proportion of all blood meals the vector population would obtain from humans in the absence of nets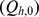. Low values of 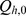 represent mosquitoes that primarily feed on animals while high values represent mosquitoes that prefer to feed on humans. The y-axis for panel **A** represents the absolute entomological inoculation rate (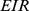) for an average community member in a given scenario 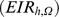. The y-axes for all other panels represents relative values for mosquito population parameters, compared with those expected in the absence of LLINs: **B**: Relative exposure for non-users, 
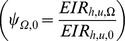
 **C**: Relative proportion of blood-meals taken from human 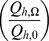, **D**: Relative probability of surviving one complete feeding cycle 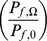, **E**: Relative feeding cycle length 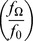, and **F**: Relative emergence rate of adult mosquitoes 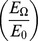. In all cases the intervention scenario 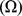 crude demographic coverage specified high levels of coverage 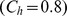 and use at times when transmission would otherwise occur 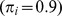.

For all panels in figure 1, equation 5 was used to plot independent x-axis values representing simulated values of the proportion of blood meals taken from humans in the absence of an intervention 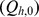. Low values of 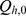 represent mosquitoes that primarily feed on animals while high values represent mosquitoes that prefer to feed on humans. The y-axis for panel **A** represents the absolute entomological inoculation rate (EIR) for average community member in which the dependent values were plotted using equation 12. The y-axes for all other panels were plotted using equations given in brackets representing relative values for mosquito population parameters when compared with those expected in the absence of LLINs: **B**: Relative exposure for non-users 
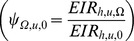
, equation 13 **C**: Relative probability of surviving one complete feeding cycle 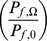 (equation 14), **D**: Relative proportion of blood-meals taken from human 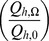, (equations 4 and 5) **E**: Relative feeding cycle length 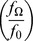, equation 15, and **F**: Relative emergence rate of adult mosquitoes 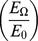 equation 16.

Consistent with field observations [4]–[5], [10], [12], [21], [48]–[51] and previous simulations, high coverage with an insecticidal personal protection interventions is predicted to have huge immediate impact on malaria transmission where mosquitoes primarily feed indoors upon humans (Figures 1 A and B). Insecticidal personal protection is most effective against human-feeding mosquitoes (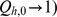 because the fraction of available blood resources that protected people represent is high so that survival per feeding cycle is reduced (Figure 1C), the length of feeding cycle is extended (Figure 1E), and the emergence rate for adult mosquitoes is reduced (Figure 1F) [6], [48], [50]–[51].

By comparison, as previously described [4]–[5], [13], insecticidal personal protection measures are less efficacious against mosquitoes that only occasionally feed upon humans (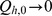) because animals are not protected and remain available to feed on. Therefore, negligible impact is expected upon mosquito survival equation 14, Figure 1C or upon feeding cycle length equation 15 Figure 1E, or upon reproduction rates equation 16, Figure 1F. Human blood index is the only parameter affected for very zoophagic vectors (Figure 1D) so it is important to explore what happens to 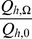 as 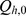 approaches zero.

Personal protection measures can deliver appreciable communal protection against transmission by zoophagic vectors (Figure 1B) because they can lower the proportion of bloodmeals obtained from humans (Figure 1D). Thus, further reducing already-low proportions of blood meals taken from humans (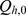), can have a corresponding immediate impact on the exposure of non-users lacking any personal protection against malaria transmission by zoophagic mosquitoes (Figure 1D). This is because the tiny proportion of a zoophagic mosquitos population that are killed may be a large proportion of those that actually transmit human parasites such as *Plasmodium falciparum* and *P. vivax*.

### Calculating Immediate Impact of Personal Protection Upon Transmission by Very Zoophagic Vectors using only Three Input Parameters

Next, we illustrate how the dependence of transmission and control enables derivation of much simpler models for both immediate and delayed impacts (with and without assuming reduced human infectivity, respectively) upon malaria transmission, to be derived that rationalize the reduced, but nevertheless useful, impacts of a personal protection measure upon zoophagic vector systems that are illustrated by the intercepts on the left hand side of Figures 1B and D.

**Figure 2:**
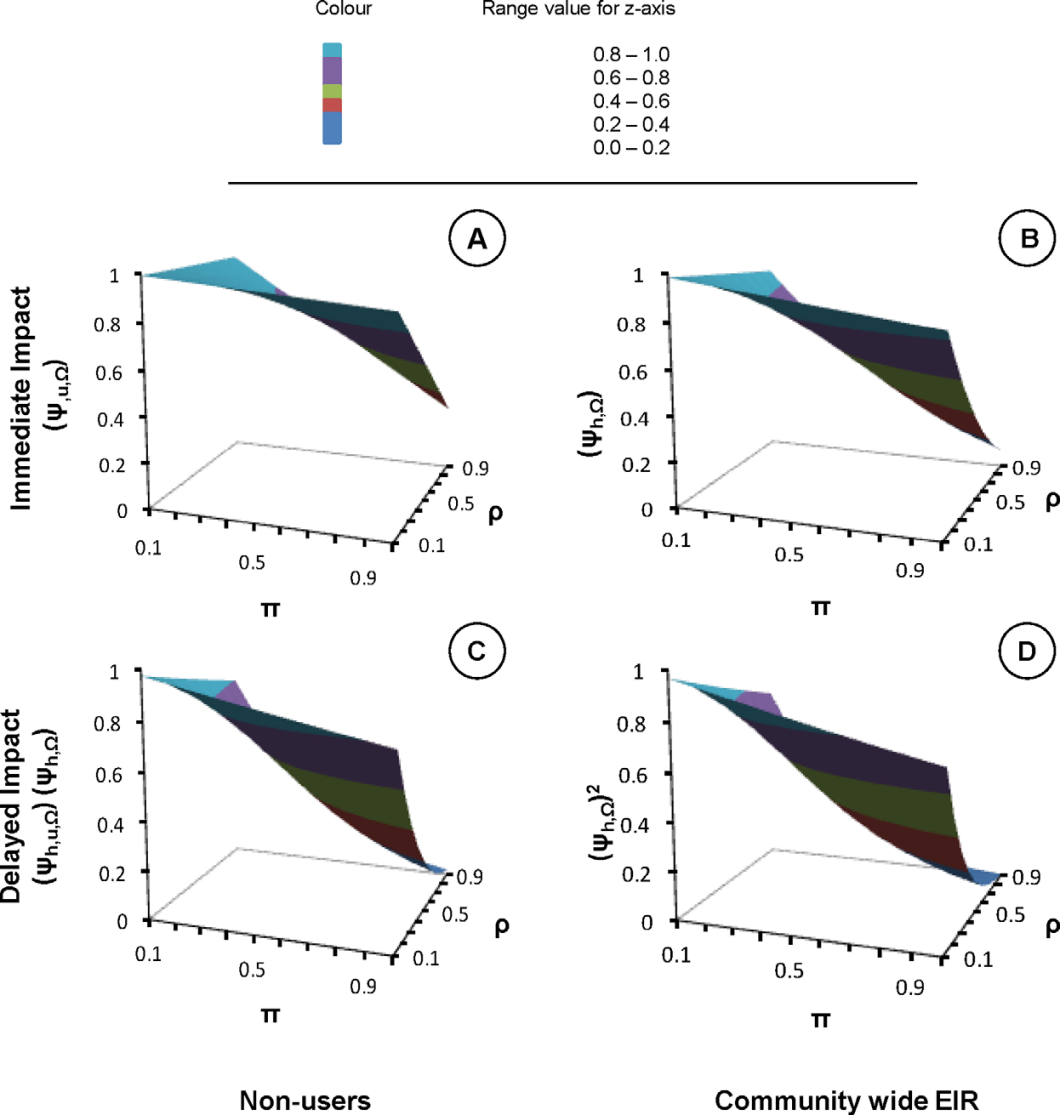
Immediate and delayed impact of personal protection upon malaria transmission intensity. In all the four panels, x-axis is the proportion of human exposure to mosquito bites that would otherwise occur when the protective intervention is used 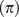 and y-axis represents the proportion of mosquito bites prevented by using that protective intervention 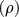. The z-axes reflects immediate (**A** and **B**) and delayed (**C** and **D**) relative exposure 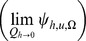 experienced by non-users (**A and C**) and average community members (**B** and **D**).

So, as 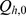 approaches zero, the immediately relative exposure of non-users benefiting only from communal protection (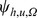) (Figure 2B), compared to their pre-intervention exposure can be computed as follows; If we substitute equation for 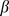 and 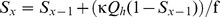, into equation 13 we get;
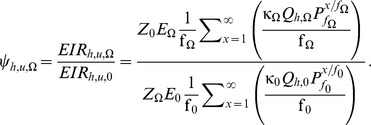



By assuming that 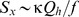 on the basis that sporozoite rates are proportional to 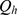 and therefore very low for very zoophagic vectors so a mosquito only gets one chance to get infected, and if we take out all terms not affected by 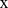 out of summation and rearrange then;
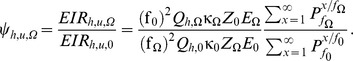



We assume that 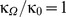 in the short term because substantive changes in human infection prevalence take months or years [52]–[53]. We know that by taking a limit as 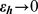, 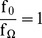 equation 15, 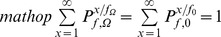 (see steps in equation (16)), 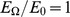 and 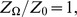 since 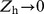 as 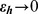, then 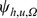 is given by;
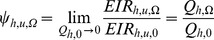
(17)


Now, if we substitute the definition of 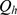 from equation 4, rearrange and substitute 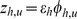 and 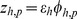 where 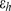 is human encounter rate [6], relative exposure of non-users (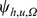) is intuitively calculated as the mean of the feeding probabilities for protected 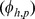 and unprotected humans 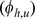, weighted according to the protective 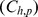 rather than simple demographic 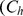) coverage:
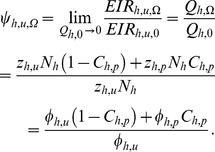
(18)


In simple terms, the level of indirect communal protection afforded to all community members is equivalent to the coverage-weighted mean of feeding probabilities (equation 18). This is, in turn, equivalent to the community-wide mean level of person protection obtained as a coverage-weighted mean of personal protection. Relative exposure can also be expressed in terms of personal protection 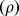, where [6];
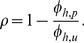
(19)


So, by substituting equations 1 and 19 into rearranged equation 18, the impact upon transmission by very zoophagic vector can be expressed in terms of only three field-measurable parameters: the proportion of human exposure to mosquitoes occurring when an intervention can be practically used (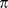), its protective efficacy when used 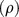, and the proportion of people using it (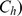:
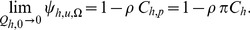
(20)


Of course communal protection is complemented by personal protection so the overall mean level of protection immediately obtained across all users and non-users in the community is calculated as the square of equations 18 and 20. Consistent with previous models [6], [8], [36], [50]–[51], [54]–[56], the immediate relative exposure of the average community member (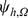) is equivalent to the ratio of the square of the pre and post intervention human blood index (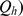 values.
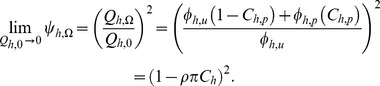
(21)


In direct, intuitive terms, this is because a mosquito has to bite humans twice to transmit malaria parasites.

### Delayed Impacts Including Reduced Human Infectiousness

The relatively low transmission intensities that very zoophagic mosquitoes mediate, also allow the reduction of infectiousness of the human population to mosquitoes to be approximated in a simplified manner. In addition to the direct and immediate impacts upon the vector population, reduction impacts upon infectiousness of human population to mosquitoes 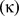 may also be achieved [31], [52] but only if mosquito to human transmission can be reduced below saturating levels 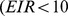 infectious bites per person per year) [57]. In holoendemic scenarios, with highly anthropophagic vectors, getting below this threshold will require high levels of coverage 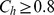 over long periods because re-equilibration of transmission and prevalence levels will take years rather than days, weeks or months [52], [58]. At the expected intermediate levels of residual transmission 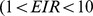 infectious bites per person per year) expected for anthropophagic vector populations exposed to high intervention coverage (Figure 1A), the eventual impact upon EIR, resulting from direct immediate impact on the vector population parameters combined with feedback upon human infectiousness is complex to predict [57]–[59].

While human infectiousness is saturated at high transmission levels (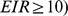, at the much lower levels expected for most very zoophagic vectors 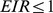, human infectiousness to mosquitoes is thought to be directly and approximately linearly related to mosquito to human transmission intensity in the previous few years 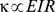. While impacts upon the vector population have an immediate effect on _EIR_ (Figure 2A), no immediate impact upon infectiousness is expected (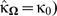 and it may take a long time for a long-lived blood stage infection to be cleared from the human population and the feedback of _EIR_ upon 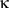 and *vice versa* to re-equilibrate [49]–[50]. Assuming a linear relationship exists between these two variables at low values approaching the origin of Figure 1A, and that further reductions will be achieved as a result of re-equilibration between 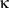 and 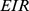, then reduction of impact on human infectiousness to mosquitoes is expected to be greater than the immediate impact on EIR.
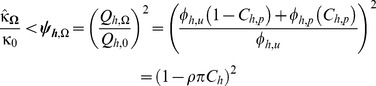
(22)


The combination of effects mediated by the immediate impact on vector population, and delayed impact on malaria parasite prevalence and mean infectiousness in the human population, is therefore assumed to at least the same as the product of the two:
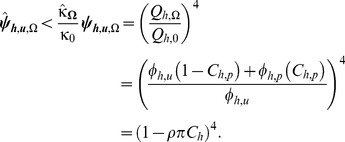
(23)


The most obvious implication of these simplified models is captured directly in equations 18 and 20. For very zoophagic vectors, overall impact is directly related to efficacy of personal protection, regardless of whether that arises from deterrent or toxic models of action. The only other primary determinants are crude coverage 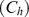 and the proportion of non-user exposure occurring when the protective measure can practically be used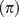.

### Thresholds Necessary to Attain Epidemiological Impact

In all the panels of figure 2, the x-axis is the proportion of human non-user exposure to mosquito bites that occurs at times when a user would actually use the protective intervention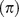, which was plotted in values decreasing from 0.9 to 0.1 in the interval of 0.1. The y-axis represents the proportion of mosquito bites prevented while actually using protective intervention obtained by taking the product of 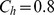 and the values from equation 19.The z-axes reflects immediate (**A** and **B**) and delayed (**C** and **D**) impact upon relative exposure experienced by non-users. While the latter assumes that delayed effects upon human-to-mosquito transmission occur if immediate reductions in the ability of mosquitoes to mediate transmission to humans are sustained over a long time [52]. Therefore, figure 2 is produced as follows; the x-axis in all panel are 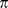 values decreasing from 0.9 to 0.1, the y-axis are calculated protective 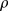 values from the given expression. In other hand, a different equation was used for each panel to obtain values for z-axis by using corresponding 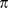 and protective 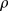 values substituted into equation 20 (**A**), equation 21 (**B**), product of values from equations 20 and 21 (**C**) and equation 23 (**D**).

In figure 2, the reader can note that the values in z-axes only start dropping substantially at higher values of the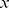 and 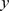 axes. Thus, figure 2 illustrates how these simplified models indicate that personal protection measures will need to be practically applicable at most times of the day when exposure can occur 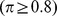, confer high levels of person protection to users 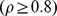, and be used by the majority of human population 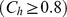, if they are to appreciably suppress malaria transmission by zoophagic vectors.

## Discussion

Human blood index, defined as the proportion of a mosquito population that feeds upon humans, is clearly as important a determinant of malaria transmission and control (Figure 1) today [29] as it was half a century ago [19]. In simple terms, the more a vector depends upon human blood, the greater will be the impact of personal protection measures upon their population density, longevity and transmission potential, and the greater will be the advantage of pesticides which act exclusively through contact toxicity over those relying upon repellency (Figure 1). However, the more zoophagic a mosquito species is, the more personal protection can act simply by blocking host-vector contact (Figure 1) so that it becomes increasingly irrelevant whether protection is achieved through toxicity or repellency so that a wider variety of target product profiles may be considered [60].

The world’s malaria vectors span the full range of baseline human blood indices considered here [17], [19] so this remains a critical parameter for national control programmes to evaluate and consider when planning vector control campaigns. The findings from the models presented apply specifically to very zoophagic vectors, mosquitoes with a strong preference for animals which normally obtain less than 10% of their blood meals from humans, but may still mediate malaria transmission. While the simplified models developed here only apply in settings where a purely anthroponotic pathogen is transmitted by a predominantly zoophagic vector, this counterintuitive situation is remarkably wide spread and important. Approximately 40% of all *Plasmodium falciparum* infections [61] and 95% of *Plasmodium vivax* infections [62] occur outside of sub-Saharan Africa, largely in parts of Asia where a wide diversity of primary vectors predominantly feed on animals rather than humans [17]. This extreme scenario contrasts starkly with the anthropophagic vectors, such as *An. gambiae*, *An funestus* and *An koliensis*, that have dominated the thinking behind global malaria control policy [8], [63]–[64]. However, it is important to note many of the most important species in residual transmission systems, such as *An. arabiensis* Africa and *An. farauti* in the Pacific, are both zoophagic and anthropophagic so that they sit between these two extremes. Surveys of human blood indices, or underlying host preference indices such as relative availability [27], [33], relative attack rates [65], or feeding indices [66]–[67] should therefore be considered as an important indicator in national entomological monitoring systems.

Where such surveys confirm very low human blood indices, the minimum immediate (equation 21) and delayed (equation 23) impacts of a personal protection measure upon transmission by very zoophagic mosquitoes can be approximately calculated with very simple models. These models use only three parameters which may potentially be measured in the field by National Malaria Control Programmes (NMCPs) and their supporting national institutional partners in developing countries: the maximum proportion of human exposure to mosquitoes that can be directly prevented through personal protection by using a given intervention, its protective efficacy when used, and the demographic coverage of human users. The relationship between entomologic inoculation rate (EIR) which is a direct, field-measurable indicator of human exposure to bites of mosquitoes infected with transmissible sporozoite stage malaria parasites [30]–[31] and the efficacy of a personal protection measure was derived through a model that logically describe the process of mosquito feeding cycle and malaria transmission.

The suggestion that the impact of personal protection upon malaria transmission by very zoophagic vectors may be independent of the mode of action of the product has substantial implications for manufacturers and NMCPs alike. Unlike transmission mediated by anthropophagic vectors [6], [60], the impact upon malaria where zooophagic vectors predominate is a simple function of personal protective efficacy regardless of whether that arises from deterrent or toxic modes of action. Vapor-phase repellents [68]–[71] do not require direct physical contact with target insects. They can protect one or more individuals without comprehensively treating wall, roof, net, clothing or skin surfaces, so high levels of personal protection may be easier to achieve in practice [60] than with the contact toxins that are clearly superior for vectors that feed indoors upon humans [6]. Such spatial repellents may therefore be particularly applicable, and even preferable to contact toxins, where malaria transmission is predominantly mediated by very zoophagic vectors, especially where transmission primarily occurs outdoors. While we present initial modeling results here, further empirical field testing of this model is essential to build solid evidence to guide malaria control programs.

### Conclusion

We extended a published malaria transmission model to examine the relationship between transmission, control, and the baseline human blood index for very zoophagic vectors. The results from model is very simple and can be used by vector control practitioners to forecast the likely immediate and delayed impacts of personal protection measures using three parameters that may potentially be measured in the field: the proportion of human exposure to mosquitoes occurring when a intervention can be practically used, its protective efficacy when used, and demographic coverage of human users. High levels (≥80%) of protective coverage and efficacy are important to achieve an epidemiologically meaningful impact.
